# One-Dimensional Nanostructures of Polypyrrole for Shielding of Electromagnetic Interference in the Microwave Region

**DOI:** 10.3390/ijms21228814

**Published:** 2020-11-21

**Authors:** Robert Moučka, Michal Sedlačík, Hayk Kasparyan, Jan Prokeš, Miroslava Trchová, Fatima Hassouna, Dušan Kopecký

**Affiliations:** 1 Centre of Polymer Systems, Tomas Bata University in Zlín, 760 01 Zlín, Czech Republic; moucka@utb.cz; 2 Department of Production Engineering, Faculty of Technology, Tomas Bata University in Zlín, Vavrečkova 275, 760 01 Zlín, Czech Republic; 3 Faculty of Chemical Engineering, University of Chemistry and Technology, Prague, 166 28 Prague 6, Czech Republic; hayk.kasparyan@vscht.cz (H.K.); fatima.hassouna@vscht.cz (F.H.); 4 Faculty of Mathematics and Physics, Charles University, 180 00 Prague 8, Czech Republic; jprokes@semi.mff.cuni.cz; 5 Central Laboratory, University of Chemistry and Technology, Prague, 166 28 Prague 6, Czech Republic; trchovam@vscht.cz

**Keywords:** conducting polymers, electromagnetic shielding, 1D nanostructures, thermal stability, microwave region

## Abstract

Polypyrrole one-dimensional nanostructures (nanotubes, nanobelts and nanofibers) were prepared using three various dyes (Methyl Orange, Methylene Blue and Eriochrome Black T). Their high electrical conductivity (from 17.1 to 60.9 S cm^−1^), good thermal stability (in the range from 25 to 150 °C) and resistivity against ageing (half-time of electrical conductivity around 80 days and better) were used in preparation of lightweight and flexible composites with silicone for electromagnetic interference shielding in the C-band region (5.85–8.2 GHz). The nanostructures’ morphology and chemical structure were characterized by scanning electron microscopy, Brunauer–Emmett–Teller specific surface measurement and attenuated total reflection Fourier-transform infrared spectroscopy. DC electrical conductivity was measured using the Van der Pauw method. Complex permittivity and AC electrical conductivity of respective silicone composites were calculated from the measured scattering parameters. The relationships between structure, electrical properties and shielding efficiency were studied. It was found that 2 mm-thick silicone composites of polypyrrole nanotubes and nanobelts shield almost 80% of incident radiation in the C-band at very low loading of conductive filler in the silicone (5% *w/w*). Resulting lightweight and flexible polypyrrole composites exhibit promising properties for shielding of electromagnetic interference in sensitive biological and electronic systems.

## 1. Introduction

Electromagnetic field emitted by modern electronic devices interferes with sensitive electronic circuits or even with living organisms in a process referred to as electromagnetic interference (EMI). Most of EMI cases are relatively harmless, manifested as a low noise of instruments or dissipated as negligible heat on the cell surface. However, EMI at certain wavelength/frequencies may cause severe distortion of useful electronics signals or even damage sensitive electronics [1,2]. Moreover, long-term exposition to EMI may lead to serious health issues [3,4,5].

The most common solution of EMI is application of various shields onto the sources or targets of electromagnetic radiation [6]. Metallic shields have been historically proven, allowing caging or reflection of the majority of the problematic electromagnetic spectrum, but they suffer from some drawbacks such as susceptibility to corrosion, high density and associated weight or low flexibility [7]. Significant attention has therefore been paid to modern low-weight, flexible materials based on composites of organic substances (usually synthetic- or bio-polymeric matrix) and highly electrically conductive fillers (metallic, carbonaceous or polymeric nano- and microparticles) [8].

One of the most appreciated features of a conductive filler beside the magnitude of its electrical conductivity is its high aspect ratio. This morphology-based property is substantial for reaching the percolation threshold (macroscopic electrically conductive pathway throughout the material volume) at minimal loading (concentration) of the filler in the matrix [9]. Here, typically one- (1D) and two-dimensional (2D) materials have an advantage over bulky three-dimensional (3D) ones. Especially 1D carbon nanotubes or 2D graphene are currently being massively tested in EMI applications. However, the range of 1D and 2D conducting fillers is not limited to the carbonaceous or metallic particles only [10]. Conducting polymers, especially polypyrrole (PPy), polyaniline and their nanostructures, represent an extraordinary group of materials with high electrical conductivity and easily controlled morphology [11,12].

PPy for EMI shielding is commonly employed in globular morphology as a composite with metallic nanoparticles or carbonaceous materials [13,14]. However, PPy can be also used as a plain powder [15,16] or structure-forming material, e.g., epoxide composites [2], film-forming material on the surface of various objects, e.g., sawdust [17,18], silver sponges [19], zeolites [20], fabrics [21,22,23,24,25], wool [26] and membranes [27]. The disadvantage of applying the globular morphology in EMI shielding is the necessity of using high loadings (~tens of % *w/w*) in order to reach the percolation threshold [16], which may result in deterioration of the mechanical properties of the used matrix.

Recently, 1D nanostructures (helical nanotubes and fibers [28], nanorods [29]) of PPy were applied in EMI shielding and exhibited promising properties. In our previous work [30], we tested and compared EMI shielding properties of several PPy 1D nano- and 3D microscaled morphologies including globules, microbarrels and nanotubes. The results confirmed superior EMI shielding properties of PPy nanotubes (*S*_21_ = −13.27 dB; equal to almost 80% of incident radiation) over PPy globules at relatively low loading (5% *w*/*w*). Moreover, PPy nanotubes exhibit higher long-term stability of their electrical properties [31], which is crucial for their application in real conditions. The PPy nanotubes used in our previous work were prepared by a popular method using azo-dye Methyl Orange as a soft template [11]. However, PPy 1D nanostructure preparation is not limited to this particular azo-dye only. Recently published articles [32,33] have shown that other dyes can act as a support for the formation of 1D structures of PPy, e.g., Acid Blue, Acid Red 1, etc.

The mechanism of synthesis of nano- and microstructures of PPy using dyes is still not understood in full extent. However, it is believed that three phenomena may play a role during PPy structure formation, depending on the type of dye used in the reaction [34]. The first one, soft nuclei formation, is based on the fact that the template-forming dye contains hydrophilic and hydrophobic parts, forming a micelle-like supporting structure in an aqueous solution. The second mechanism presumes the existence of hard nuclei formed by an insoluble product of a dye and another substance (typically an oxidant) or due to the acid–salt transition of the dye caused by pH change (again due to the presence of an acidic oxidant) in the reaction solution. Nuclei, regardless if soft or hard, are initiators of subsequent structure growth and their shape and size determine the final diameter and inner shape of the PPy structure. Finally, the third theory assumes the ability of conducting polymers to grow on the surface or in the inner spaces of objects submerged into the reaction solution. Here, the template determines the diameter, length and shape of the final PPy structure.

In the presented work, three promising candidates of PPy 1D structures with high aspect ratio for EMI shielding application in the region from 5.85 to 8.2 GHz were experimentally compared. They were synthesized in the presence of Methyl Orange (PPy-MO), Methylene Blue (PPy-MB) and Eriochrome Black T (PPy-EB) dyes. Novel nanostructures of PPy-MB and PPy-EB are results of recent research effort on the design and tuning of PPy morphology using various dyes [12]. Prepared PPy nanostructures differ from each other namely in the magnitude of their electrical conductivity (from 17.1 to 60.9 S cm^−1^) and geometrical dimensions (diameters vary from 100 to 4000 nm). The aim of our work was to find correlation among electrical conductivity, the aspect ratio of various PPy 1D structures, material preparation and its shielding efficiency. In addition, also for the first time, their structural morphological (by scanning electron microscopy—SEM, Brunauer–Emmett–Teller method—BET), dynamical (by attenuated total reflection Fourier-transform infrared spectroscopy—ATR-FTIR) and electrical properties (AC and DC electrical conductivity, complex permittivity) have been compared. These results may be subsequently used for the preparation of advanced composites intended for EMI shielding on the basis of 1D PPy structures.

## 2. Results and Discussion

### 2.1. Morphology, Dynamical and Electrical Properties

**Figure 1:**
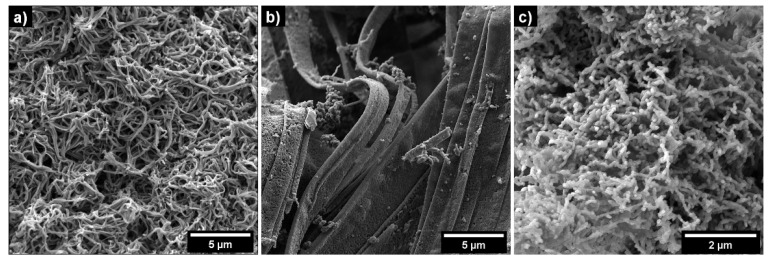
The morphology of prepared polypyrrole (PPy) powders: (**a**) Methyl Orange (PPy-MO) nanotubes, (**b**) Methylene Blue (PPy-MB) nanobelts, (**c**) Eriochrome Black T (PPy-EB) nanofibers.

The polymerization of pyrrole by iron(III) chloride hexahydrate oxidant in the presence of all three dyes yielded black crude powders indistinguishable on a macroscopic level. However, detailed microscopic SEM analysis (Figure 1) revealed substantial differences between the samples. The synthesis route using Methyl Orange as a soft-template led to the expected nanotubes having approximately 100–400 nm in diameter and units of µm in length. These 1D structures were hollow with an approximate internal diameter of 50 nm as measured in our previous work with transmission electron microscopy [34]. Both synthesis routes using Methylene Blue and Eriochrome Black T were modified compared to the recently published paper of Stejskal et al. [12]. The goal of this modification was to obtain samples with high electrical conductivity, comparable to that of Methyl Orange and with high content of 1D nanostructures at the same time.

The synthesis of PPy in the presence of Methylene Blue resulted in nanobelt morphology with diameter of approximately (in this case, width) 600–4000 nm and tens of µm in length. The synthesis in the presence of Eriochrome Black T led to the small, uniform nanofibers having approximately a hundred of nm in diameter and hundreds of nm in length.

BET measurement (Table 1) of PPy-MO revealed a specific surface area of 47.81 m^2^ g^−1^, which is almost twice the size of the specific surface area of PPy-MB (24.41 m^2^ g^−1^) and PPy-EB (27.38 m^2^ g^−1^). A surprisingly low specific surface area of PPy-EB nanofibers, the smallest 1D nanostructures, was probably given by the absence of hollow cavities.

The measurement of electrical conductivity (Table 1) revealed the highest value in the case of PPy-MO (60.9 S cm^−1^). Again, PPy-EB nanofibers, contrary to expectation, exhibited the smaller electrical conductivity (17.1 S cm^−1^), similar to that of PPy-MB nanobelts (21.5 S cm^−1^). The explanation lies probably in better arrangement of PPy chains in PPy-MO nanotubes, i.e., on the supramolecular level, whereas PPy-EB is structurally closer to the low conductive globular PPy [35]. However, the difference in electrical conductivity between prepared 1D nanostructures is in general low compared to PPy 3D structures and globular PPy (both have order of magnitude lower conductivity; around units of S cm^−1^) [33]. The measurement of stability of electrical conductivity after 80 days revealed that this time period can be considered almost equal to the conductivity half-time of PPy-MB (12.4 S cm^−1^) and PPy-EB (8.9 S cm^−1^). From this point of view, electrical conductivity of PPy-MO after 80 days (52.0 S cm^−1^) was still relatively high. The conductivity half-time of PPy-MO is around 160 days, whereas for globular PPy it is approximately 7 days [31].

**Figure 2:**
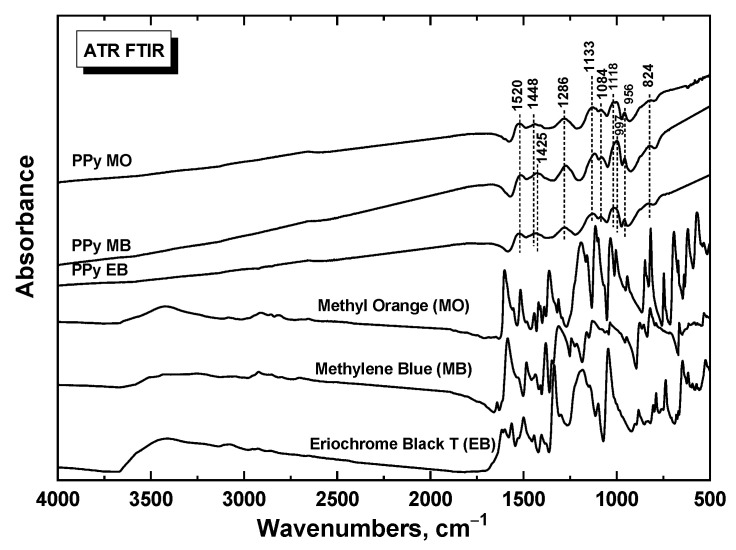
FTIR spectra of prepared samples and initial dyes.

The model of PPy nanotubes assumes that PPy chains grow during the polymerization of pyrrole from a template composed of a dye [36]. ATR-FTIR spectroscopy is a convenient technique to study the molecular properties of the polymer chains on the surface of dye template (Figure 2). The infrared spectra of PPy prepared in the presence of all three dyes correspond to the conducting state of PPy, and they are close to each other. They exhibit the main bands of polypyrrole nanotubes situated (in case of MO dye) at 1538 cm^−1^ (C–C stretching vibrations in the pyrrole ring), at 1450 cm^−1^ (C–N stretching vibrations in the ring), at about 1293 cm^−1^ (C–H or C–N in-plane deformation modes), at 1144 cm^−1^ (breathing vibrations of the pyrrole rings) and at 1027 cm^−1^ (C–H and N–H in-plane deformation vibrations) [11]. The small shifts of the main bands in the case of MB and EB dyes corresponded to their lower conductivities (Table 1). The presence of dyes does not manifest itself in the spectra when surface-sensitive ATR technique is used.

Overall, all three synthesized PPy 1D nanostructures exhibited favourable features for further application in EMI shielding. They had clean, relatively homogeneous morphology without substantial presence of impurities in the form of globular PPy. 1D shape with a high aspect ratio enables one to achieve a low percolation threshold. More importantly, their electrical conductivity was far above the average of electrical conductivity of globular PPy, which is the most frequent PPy morphology used in EMI shielding [9,10]. Finally, larger specific surface is beneficial for better compatibilization of PPy with the matrix.

### 2.2. EMI Shielding Properties

All three PPy 1D nanostructures were mixed with silicone at various loadings (1, 3 and 5% *w/w*) and subsequently cured at 25 or 150 °C. The higher temperature of curing (150 °C) simulates blending conditions for some matrices with high melting temperature. Our previous research has shown that higher processing temperature may lead to undesirable fast deterioration of electrical conductivity of PPy [31].

**Figure 3:**
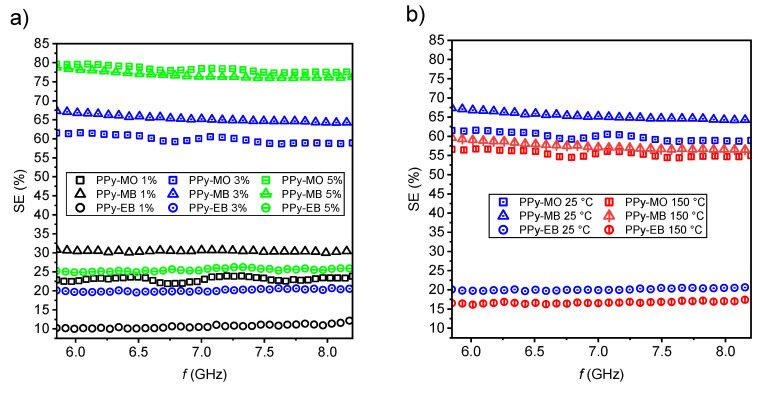
EMI shielding efficiency of PPy 1D nanostructures in the range from 5.85 to 8.2 GHz (**a**) for various loadings 1, 3 and 5% *w/w* (curing temperature 25 °C), (**b**) for curing temperatures 25 and 150 °C (loadings 3% *w*/*w*).

The shielding efficiency of all three 1D nanostructures is depicted in Figure 3a. Several trends can be observed from the calculated data. First, higher PPy loading led generally to higher shielding efficiency. The PPy-MO and PPy-MB samples shielded about 80% of the incoming electromagnetic signal (by a combination of reflection and absorption) at the concentration of 5% *w/w*. This is quite a promising result, as a commonly used loading of globular PPy is around tens of % *w/w* in order to obtain similar results [16].

**Figure 4:**
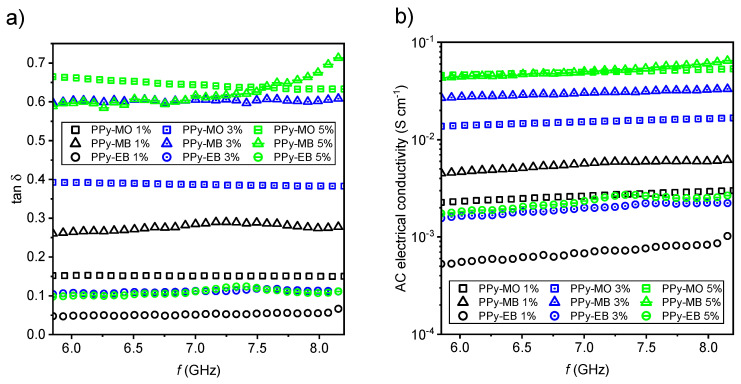
(**a**) Dielectric loss tangent of prepared PPy 1D structures and (**b**) related AC electrical conductivity (samples prepared at 25 °C, various loadings).

Both PPy-MO nanotubes’ and PPy-MB nanobelts’ morphologies exhibited similar shielding efficiency, regardless of their different electrical conductivities. The discrepancy in shielding efficiency between PPy-MB nanobelts and PPy-EB nanofibers with similar electrical conductivities stems from their different morphology, namely, their different aspect ratios. PPy-EB nanofibers have the smallest aspect ratio from all three 1D nanostructures. A high aspect ratio is a crucial feature of 1D nanostructures applied in nonconductive matrices and it may have a bigger impact on the final shielding efficiency than the absolute value of electrical conductivity [37]. PPy-EB nanofibers at 5% *w*/*w* loading was probably still below its percolation threshold, as is also indicated by low AC electrical conductivity of the respective composite (Figure 4b).

Elevated curing temperature of 150 °C had a lower impact on EMI shielding efficiency (of all the samples) than expected (Figure 3b). Shielding efficiency generally decreased by about 5%, which is still acceptable. However, the necessity of using even higher curing temperatures can be a limiting factor in future applications.

**Figure 5:**
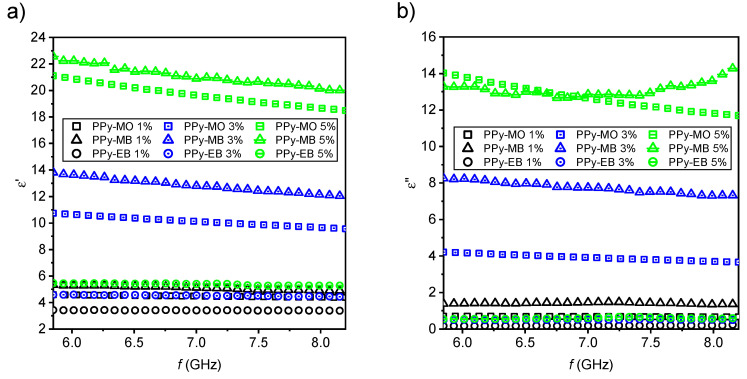
Real (**a**) and imaginary (**b**) parts of complex permittivity of PPy 1D samples prepared at 25 °C, various loadings.

PPy-MO and PPy-MB composites exhibited high values of both real and imaginary part of complex permittivity (Figure 5a,b). Calculated dielectric loss tangents (Figure 4a) indicated their low-loss character of good dielectrics (tan *δ* < 1) approaching lossy propagation materials (tan δ ≈ 1). On the contrary, PPy-EB composites (tan *δ* → 0) had low-loss character.

The final evaluation of PPy-MO, PPy-MB and PPy-EB EMI shielding properties was completed by calculated AC electrical conductivities of respective composites (Figure 4b). The highest AC electrical conductivity (of the order of 10^−1^ S cm^−1^) was achieved in the case of 1D structures, which had high aspect ratio and high loading in silicone at the same time. On the opposite side there were composites with low loading and low aspect ratio filler. AC electrical conductivity of these samples was lower by two orders of magnitude (of the order of 10^–3^ S cm^−1^).

**Figure 6:**
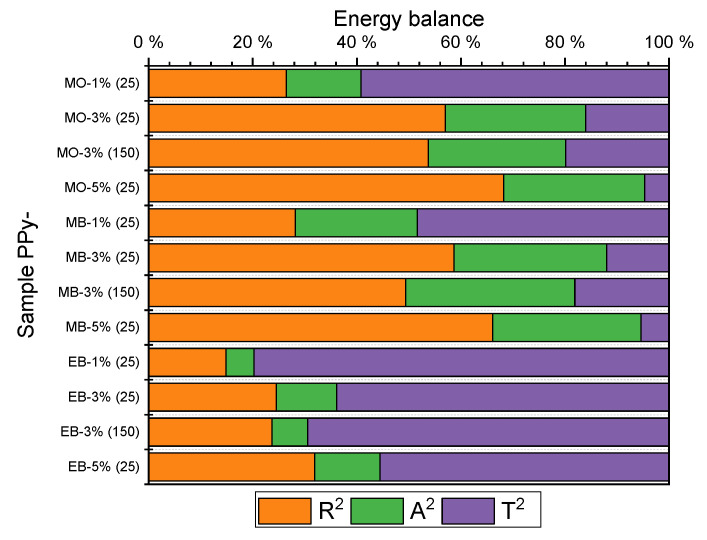
RAT (reflection, transmission, absorption) analysis of PPy 1D samples (sample curing at 25 and 150 °C, various loadings 1, 3 and 5% *w*/*w*).

Figure 6 depicts another important characteristic of PPy-MO, PPy-MB and PPy-EB composites. The so-called RAT (reflection, transmission, absorption) analysis gives information about the ability of a composite to attenuate EMI by reflection or absorption. The real applications strictly distinguish the way in which EMI is shielded off. Electromagnetic waves are reflected off the surface of a shield in the case of a prevailing reflection component (e.g., most of metals), which results in unwanted secondary EMI. The main advantage of nanostructures lies in generally higher absorption, preventing secondary EMI [7]. 

Figure 6 clearly shows that both PPy-MO and PPy-MB recorded significant absorption in the tested C-band with values around 20% for nearly all loadings and curing temperatures. On the other hand, the absorption of PPy-EB composite was almost negligible. The absorption of PPy-MO and PPy-MB was not affected by the higher curing temperature (150 °C); the 5% decrease in shielding efficiency, discussed above, was owing to the decrease in reflection. This indicates that morphology of both PPy-MO and PPy-MB was less affected by elevated temperature than electrical conductivity was.

## 3. Materials and Methods

### 3.1. Chemicals

**Figure 7:**
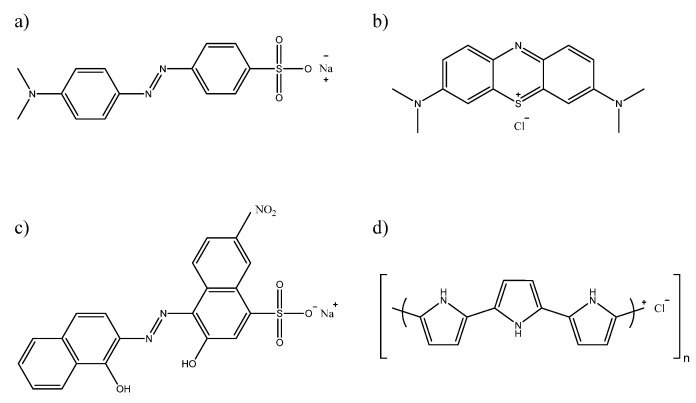
Chemical structural formulas of (**a**) Methyl Orange, (**b**) Methylene Blue, (**c**) Eriochrome Black T dye and (**d**) polypyrrole.

Monomer: pyrrole (98%, Sigma-Aldrich); dyes: Methyl Orange (dye content 85%, Sigma-Aldrich, Missouri, USA, Figure 7a), Methylene Blue (indicator grade, Lachema, Czech Republic, Figure 7b), Eriochrome Black T (indicator grade, Lachema, Figure 7c); oxidant: iron(III) chloride hexahydrate (99%, Sigma-Aldrich). All chemicals were used as purchased. All reactions were made in a distilled water environment. A Sylgard 184 kit including a catalyst (Dow Corning) was used as a silicone matrix for PPy dispersion.

### 3.2. Synthesis of 1D Structures of Polypyrrole 

The PPy-MO nanotubes were prepared in the presence of Methyl Orange azo-dye according to previously published procedure [35]. Briefly, 0.03 mol of pyrrole (2.09 mL) was dissolved in a 2.5 M solution of Methyl Orange (600 mL). 0.03 mol of iron(III) chloride hexahydrate (8.12 g) was dissolved in distilled water (69 mL). Oxidant solution was drop-wisely added into the mixed pyrrole solution. Hence, the molar ratio of polymer to oxidant was 1:1. The temperature of synthesis was kept at 5 °C and the duration of polymerization reaction was 24 h. The obtained black powder was purified in 2-day long Soxhlet extraction using acetone. Finally, dye-free PPy was rinsed with ethanol, dried and homogenized using a pestle and mortar. 

PPy-MB nanobelts and PPy-EB nanofibers were prepared by modification of a recently published procedure in order to obtain higher electrical conductivity [12]. 

0.01 mol of pyrrole (0.69 mL) was dissolved in 0.02 M solution (50 mL) of Methylene Blue and 0.01 M solution (50 mL) of Eriochrome Black T, respectively. The solution was tempered at 5 °C and subsequently, 0.025 mol of iron(III) chloride hexahydrate dissolved in distilled water (50 mL) was added. The molar ratio of polymer to oxidant was 1:2.5. The reaction proceeded in a cooled bath at 5 °C for 24 h under vigorous stirring. The final black powders were filtered and rinsed using a 0.2 M solution of HCl (25 mL), distilled water (1.5–2 L) and ethanol (100 mL). Dried samples were homogenized using a pestle and mortar. All the samples were stored in a plastic box not exposing them to sunlight at room temperature.

### 3.3. Characterization of 1D Structures by Electron Microanalysis

An 8–10 nm-thick carbon layer, for prevention of charging during electron microanalysis, covered all the samples (carbon coater Leica EM ACE600, Leica Microsysteme, Germany). A Schottky-cathode (3 kV of accelerating voltage) scanning electron microscope MIRA 3 LMH (Tescan company, Czech Republic) was used for morphology investigation of the prepared samples.

### 3.4. Specific Surface Measurement by Brunauer–Emmett–Teller Analysis

The specific surface area of the PPy powders was evaluated by nitrogen physisorption at 77 K using a Belsorp-mini II (BEL Japan, Inc.) device. Prior to adsorption, the samples were outgassed for 17 h at 140 °C in a vacuum. Multipoint Brunauer-Emmett-Teller analysis of resulting nitrogen adsorption isotherms was carried out in BEL Master software (version 6.4.1.0, MicrotracBEL Corp.).

### 3.5. Measurement of DC Electrical Conductivity

Room temperature conductivity of the low-resistance samples was determined using the four point contacts method described by van der Pauw. The measuring setup was based on Keithley 220 Programmable Current Source, Keithley 2010 Multimeter as a voltmeter and a Keithley 705 Scanner equipped with a Keithley 7052 Matrix Card. For high-resistance samples, the measuring setup was modified as described in [31].

### 3.6. Preparation of Samples for EMI Shielding Measurement

For EMI shielding measurement, three sets of composite samples were prepared, each of which comprised a corresponding filler (PPy-MO, PPy-MB, PPy-EB) thoroughly dispersed by mixing in a commercial polydimethylsiloxane (PDMS) Sylgard 184 kit. Each set of composites contained three different concentrations, namely 1, 3 and 5 wt%. From the prepared mixtures, air was being removed for 15 min in low-pressure atmosphere (10 mbar) and subsequently, the mixtures were cast into 2 mm-thick Teflon moulds to shape them into plates. Samples were cured at two different temperatures, ambient and 150 °C, for 48 h and 20 min, respectively, in order to confirm that elevated temperature did not negatively affect electrical conductivity of PPy. Once cured, rectangular samples (35 × 16 mm) for EMI measurement in the waveguide were cut out.

### 3.7. Study of structure by Infrared Spectroscopy

ATR-FTIR spectra were recorded using a Thermo Nicolet 6700 FTIR Spectrometer equipped with a GladiATR (PIKE Technologies, Madison, WI, USA) using an ATR monolithic diamond for the full range of 4000–400 cm^−1^ with a DLaTGS (deuterated L-alanine doped triglycine sulfate) detector. Typical parameters used: 64 sample scans, resolution 4 cm^−1^, Happ–Genzel apodization, KBr beamsplitter. The spectra were corrected for the carbon dioxide and humidity in the optical path.

### 3.8. Measurement and Evaluation of Shielding Efficiency

Electromagnetic interference shielding properties of the studied composites were measured using a PNA-L network analyser (Agilent N5230A) and a rectangular waveguide (WR 137) in the frequency range from 5.84 GHz to 8.2 GHz (C-band). Rectangular composite samples were inserted into the waveguide, completely filling in its cross-section and intensity, as well as the phase of incident (*I*_0_) and transmitted (*I*_T_) or reflected (*I*_R_) electromagnetic signal, was measured, from which scattering parameters (*S*_21_ and *S*_11_) were obtained.

(1) $$S_{21}\left(dB\right)=20\;\log\frac{I_{T}}{I_{0}}$$

(2) $$S_{11}\left(dB\right)=20\;\log\frac{I_{R}}{I_{0}}$$

*S*_11_ equals to the amount of reflected intensity of the incident signal while *S*_21_ equals to the portion of the signal transmitted through the measured sample.

### 3.9. Extraction of Complex Permittivity and AC Electrical Conductivity

Knowledge of both the magnitude and the phase of reflected and transmitted waves enables to extract material parameters, i.e., complex permittivity (ε* = *ε*′ − j*ε*″), of the measured samples using the Nicholson–Ross–Weir model [38,39]. The loss part of complex permittivity can be then used for the calculation of AC electrical conductivity (*σ*) of the measured material:

(3) $$\sigma \left(\omega \right)=\omega \varepsilon_{0}\varepsilon ”$$

where *ω* is angular velocity (=2πf), *ε*_0_ is permittivity of vacuum, *ε*″ is the loss part of complex permittivity.

## 4. Conclusions

PPy synthesized in the presence of various organic dyes creates uniform nanostructures of 1D, 2D or 3D morphology, exhibiting favorable properties for application in shielding of EMI. 

Here, PPy nanotubes, nanobelts and nanofibers were studied and their morphology, size, specific surface area and DC electrical conductivity were compared. Subsequently, their composites with silicone were prepared at various loadings and curing temperatures for shielding of EMI in the C-band region covering 5.85–8.2 GHz. EMI shielding properties were evaluated based on measured scattering parameters and, furthermore, complex permittivity and AC electrical conductivity of all the composites were calculated.

From the obtained results, it can be concluded that all synthesized 1D nanostructures—nanotubes, nanobelts and nanofibers—exhibit promising morphology (easy to prepare, uniform, clean, of high specific surface area) and high DC electrical conductivity (within the same order of magnitude) to be applied in EMI shielding. However, only PPy nanotubes and nanobelts prepared in presence of Methyl Orange and Methylene Blue, respectively, can be considered as good fillers for their respective silicone composites. Both PPy nanotubes and nanobelts possessed a high aspect ratio suitable for reaching the percolation threshold at low loading (3 and 5% *w/w*). Unfortunately, PPy nanofibers synthesized in presence of Eriochrome Black T failed in this application. 

The shielding efficiency of PPy nanotubes and nanobelts was similar, reaching almost 80% in the C-band region at 5% *w/w* and curing temperature of 25 °C. RAT analysis pointed at a relatively high absorption component of both materials, which was around 20%; the remaining percentage of shielding efficiency stemmed from the reflection. The higher curing temperature at 150 °C had only a minor effect on shielding efficiency, equal to an approximately 5% decrease in reflection.

In general, recently developed PPy nanostructures seem to be promising in the preparation of lightweight, easily processable and flexible EMI shields. As their morphology and electrical conductivity have been continuously and intensively developed [12], novel and even more effective PPy nanostructures can be expected soon.

## Figures and Tables

**Table 1:** BET measurements and DC electrical conductivity of PPy.

Sample	Specific Surface (m^2^ g^−1^)	Initial Powder Conductivity (S cm^−1^)	Conductivity after 80 Days (S cm^−1^)	Approx. Diameter (nm)
PPy-MO	47.81	60.9	52.0	100–400
PPy-MB	24.41	21.5	12.4	600–4000
PPy-EB	27.38	17.1	8.9	50–150
